# Entwicklung und inhaltliche Validierung eines Fragebogens für funktionelle Bewegungsstörungen

**DOI:** 10.1007/s00115-021-01247-1

**Published:** 2021-12-23

**Authors:** Rosa Michaelis, Norbert Brüggemann, Georg Ebersbach, Christos Ganos, Alexander Münchau, Tamara Schmidt, Anne Weißbach, Uwe Schlegel, Stoyan Popkirov

**Affiliations:** 1 Klinik für Neurologie, Universitätskrankenhaus Knappschaftskrankenhaus Bochum, Ruhr-Universität Bochum, In der Schornau 23–25, 44892 Bochum, Deutschland; 2 Fakultät für Gesundheit, Universität Witten/Herdecke, Witten, Deutschland; 3 Klinik für Neurologie, Universität zu Lübeck, Lübeck, Deutschland; 4 Institut für Neurogenetik, Universität zu Lübeck, Lübeck, Deutschland; 5 Neurologisches Fachkrankenhaus für Bewegungsstörungen/Parkinson, Kliniken Beelitz GmbH, Beelitz-Heilstätten, Deutschland; 6 Klinik für Neurologie mit Experimenteller Neurologie, Charité – Universitätsmedizin Berlin, Berlin, Deutschland; 7 Institut für Systemische Motorikforschung, Universität zu Lübeck, Lübeck, Deutschland

**Keywords:** Dissoziative Bewegungsstörung, Psychogen, Konversionsstörung, Endpunkt, Patient-reported outcome measure, Dissociative movement disorder, Psychogenic, Conversion disorder, Outcome measure, Patient-reported outcome measure

## Abstract

Von Kriegszitterern zu Teenies mit TikTok-Tics – funktionelle Bewegungsstörungen galten lange als motorischer Ausdruck seelischer Turbulenzen. Tatsächlich werden psychodynamische Modelle jedoch zunehmend durch neurophysiologische Erkenntnisse ergänzt, sodass die Kombination von Psychotherapie mit spezialisierter Physiotherapie an Bedeutung gewinnt. Bislang existiert allerdings kein störungsspezifisches Ergebnismaß, welches patientenrelevante Aspekte dieser vielfältigen Störung adäquat abbildet. Ein erster derartiger Fragebogen wurde entwickelt und in einem mehrstufigen Entwicklungsprozess inhaltlich validiert. Relevanz und Verständlichkeit der Items wurden zuerst durch ein Expertengremium und anschließend durch betroffene Patienten evaluiert und entsprechend angepasst. Der so konstruierte Fragebogen erlaubt erstmals eine inhaltlich valide Quantifizierung der subjektiven Beschwerden und Auswirkungen funktioneller Bewegungsstörungen. Als Nächstes erfolgt eine multizentrische teststatistische Validierung.

**Zusatzmaterial online:**

Die Online-Version dieses Beitrags (10.1007/s00115-021-01247-1) enthält weitere Infomaterialien. Beitrag und Zusatzmaterial stehen Ihnen auf www.springermedizin.de zur Verfügung. Bitte geben Sie dort den Beitragstitel in die Suche ein, das Zusatzmaterial finden Sie beim Beitrag unter „Ergänzende Inhalte“.

## Abstract (en)

From shell shock tremors to TikTok tics, functional movement disorders have long been assumed to be motor expressions of emotional turmoil. However, psychodynamic explanations are increasingly complemented by neurophysiological findings, meaning that specialized physiotherapy is gaining in importance alongside psychotherapy. Still, there is no disease-specific outcome measure that adequately assesses patient-relevant aspects of this heterogeneous condition. Such a questionnaire was developed and its content was validated in a multistage development process. The relevance and comprehensibility of the items were first evaluated by a panel of experts and then by affected patients, and questions and possible response categories were adjusted accordingly. The resultant revised questionnaire yields good content-related validity and thus allows, for the first time, a quantification of the subjective complaints and implications associated with functional movement disorders. The next step will be a multicenter study to analyze the psychometric properties and factorial structure of this new instrument.

## Einleitung

Was haben die Kriegszitterer des Ersten Weltkriegs gemeinsam mit TikTok-Usern, die unter dem Einfluss populärer Tourette-Videos Tic-artige Paroxysmen entwickeln [10, 31, 38]? Es handelt sich um funktionelle (dissoziative/psychogene) Bewegungsstörungen, die auch jenseits dieser Sonderfälle eine häufige klinische Herausforderung darstellen. In Notaufnahmen erweisen sich 20 % aller Bewegungsstörungen als funktionell [2], in Spezialambulanzen für Bewegungsstörungen sind es 10–37 % [19, 30] und auf neurogeriatrischen Stationen sind 11 % der Patienten betroffen [21]. Das klinische Bild ist dabei vielgestaltig und reicht vom Zittern und Zucken einzelner Extremitäten über persistierende Lähmungen und Fehlhaltungen bis hin zu komplexen Mischformen mit erheblicher Einschränkung der Mobilität und Selbstständigkeit.

Die Diagnosestellung soll entgegen der gängigen Praxis nicht in erster Linie durch die Erhebung psychischer Auslöser oder durch den apparativen Ausschluss ätiologisch relevanter struktureller Pathologien erfolgen, sondern durch die klinische Feststellung störungsspezifischer Charakteristika. Neben pathophysiologischen Inkongruenzen des klinischen Bilds sind somit insbesondere klinische Untersuchungsbefunde entscheidend, die eine aufmerksamkeitsabhängige Inkonsistenz der Symptome aufzeigen (z. B. Hoover-Zeichen, Tremor-Entrainment, Dual-task-Interferenz, Placeboeffekt; Übersicht in [36] und [7]).

Obwohl funktionelle Bewegungsstörungen in der ICD-10-Klassifikation als psychiatrische Diagnosen eingeordnet sind (F44.4), werden klassische psychodynamische Konzepte der Psychogenese (Konversion, Dissoziation) zunehmend durch neurophysiologische und neuropsychologische Störungsmechanismen ergänzt [6]. Funktionelle Bewegungsstörungen werden dabei auf eine Fehlanpassung des motorischen Systems zurückgeführt, bei der die Willkürmotorik aufmerksamkeitsabhängig durch Symptomerwartungen verzerrt wird. Sicherheitsverhalten (z. B. schmerzbedingt) erhöht das motorische Kontrollbedürfnis zusätzlich, was die automatisierten motorischen Abläufe überschreibt und, stattdessen, gemäß der verinnerlichten Erwartungshaltung verzerrt [36].

Therapiestudien, die eine störungsspezifische Physiotherapie als unverzichtbare Ergänzung psychotherapeutischer Methoden beinhalten [20, 27], haben auch hierzulande Therapieerfolge nachweisen können [41].

Mit der zunehmenden wissenschaftlichen Beschäftigung mit diesem Störungsbild wurde auch die Notwendigkeit einheitlicher, valider und sensitiver Erhebungsinstrumente und Verlaufsparameter erkannt [22]. Eine neue systematische Literaturrecherche zum Thema [34] identifizierte lediglich eine von Behandlern zu bewertende störungsspezifische Skala für funktionelle Bewegungsstörungen: die Psychogenic Movement Disorder Rating Scale (PMDRS; [17]), von der auch eine Kurzversion existiert (Simplified Functional Movement Disorders Rating Scale [S-FMDRS]; [25]). Das Instrument soll der systematischen Erfassung der häufig bei Betroffenen vorliegenden vielgestaltigen klinischen Phänotypen funktioneller motorischer Symptome dienen. Allerdings setzt sich im klinisch-wissenschaftlichen Kontext hierfür zunehmend die Videodokumentation motorischer Phänomene durch, die gegenüber deren ausschließlicher Erfassung in Form eines Fragebogens offensichtliche Vorteile hat [41]. Deshalb werden PMDRS und S‑FMDRS insbesondere im wissenschaftlichen Kontext – wenn überhaupt – gewöhnlich in Kombination mit einer Videodokumentation angewandt.

Funktionelle Bewegungsstörungen sind sowohl bezüglich ihrer starken inter- und intrapersonellen Variabilität als auch in Bezug auf neuropsychologische, neuropsychiatrische und vegetative Begleiterscheinungen äußerst komplex. Der Schweregrad der motorischen Störung und die Beeinträchtigung, die von ihr ausgeht, sollten daher nicht anhand punktueller Fremdbeurteilung der motorischen Fähigkeiten (sei sie noch so detailliert, objektiv und reliabel) abgebildet werden. Bislang wurde diesem Problem begegnet, indem Fragebögen zur Selbstauskunft, also „patient-reported outcome measures“ (PROM), bezüglich der gesundheitsbezogenen Lebensqualität sowie allgemeiner psychischer und körperlicher Beschwerden angewandt wurden [26]. Einige Studien setzten gar keine PROM, sondern ausschließlich durch Behandler zu bewertende Instrumente ein [8, 11], die oft keine Informationen über das Befinden der Patienten beinhalten. Dabei gilt die Einbeziehung patientenrelevanter Ergebnisparameter aus Patientensicht mittlerweile als unumstrittener Qualitätsstandard in medizinischen Interventionsstudien [18].

Bisher wurde kein Ergebnismaß entwickelt, das störungsspezifisch die subjektiv empfundene Einschränkung durch die funktionelle Bewegungsstörung mittels eines PROM erfasst. Allerdings finden sich im Vergleich zwischen Betroffenen und Untersuchern häufig deutliche Unterschiede hinsichtlich des wahrgenommenen Schweregrads der Beeinträchtigung [29]. Stärker als durch die Störung der Bewegungsfähigkeit wird die Lebensqualität vieler Patienten mit funktionellen Bewegungsstörungen durch nichtmotorische funktionelle Symptome beeinträchtigt, wie z. B. Sensibilitätsstörungen, Konzentrationsstörungen oder Fatigue [7]. Diese Beobachtungen legen nahe, dass die Einbindung der Patientensicht durch ein störungsspezifisches PROM eine notwendige Ergänzung der bisher verwendeten Erhebungsinstrumente zur Erfassung von Veränderungen funktioneller Bewegungsstörungen darstellt. Insbesondere um die Wirksamkeit verschiedener Interventionen in Zukunft besser untersuchen und vergleichen zu können, erscheint die Entwicklung eines störungsspezifischen PROM für funktionelle Bewegungsstörungen neben der Vereinheitlichung der ergänzend verwendeten Ergebnismaße unentbehrlich [34].

## Methodik

Damit störungsspezifische Beeinträchtigungen bei Patienten mit funktionellen Bewegungsstörungen zukünftig in Studien besser erfasst werden können, wurde ein Fragebogen zur Erfassung des subjektiv empfundenen Störungsschweregrads und der Alltagsbeeinträchtigung durch funktionelle Bewegungsstörungen entwickelt und in einem mehrstufigen, iterativen Prozess mit einer strukturierten Itembewertung durch ein Expertengremium und sogenannten „kognitiven Interviews“ mit Betroffenen inhaltlich validiert. Die Durchführung dieser Fragebogenvalidierung wurde von der Ethik-Kommission der Ruhr-Universität Bochum (20-7128) genehmigt; Patienten wurden über die Studie aufgeklärt und stimmten der Publikation anonymisierter Daten zu.

### Erstellung des Itempools

Die erste Zusammenstellung relevanter Items erfolgte unter Berücksichtigung der charakteristischen Ausprägung funktioneller Bewegungsstörungen [36]. Neben dem phänotypischen Spektrum und der anatomischen Lokalisation der motorischen Beeinträchtigung wurden auch charakteristische Begleitbeschwerden und wichtige Bereiche der Alltagsfunktionalität bedacht [14]. Berücksichtigt wurden auch existierende Skalen und Fragebögen zu funktionellen Bewegungsstörungen und anderen funktionellen neurologischen Störungen [34].

Bei der Erstellung der ersten Fragebogenversion auf der Basis der systematischen Literaturrecherche wurde berücksichtigt, dass bei Betroffenen häufig eine Mischung verschiedener funktioneller motorischer Symptome vorliegt und dass die Bewegungsfähigkeit im Alltag bei vielen Patienten durch zusätzliche nichtmotorisch funktionelle neurologische Symptome beeinträchtigt wird. Allerdings wurde eine Überlappung mit anderen Instrumenten, z. B. zur Erhebung der gesundheitsbezogenen Lebensqualität und psychiatrischer Begleiterkrankungen, bewusst vermieden, da der entwickelte Fragebogen diese nicht ersetzen, sondern eine sinnvolle störungsspezifische Ergänzung anderer relevanter Erhebungsinstrumente darstellen soll.

### Strukturierte Itembewertung durch Expertengremium: Berechnung der inhaltlichen Validitätsindizes

Die inhaltliche Validität der ersten Fragebogenversion wurde mittels einer strukturierten Expertenbefragung hinsichtlich Verständlichkeit (zweistufige Likert-Skala) und Relevanz (vierstufige Likert-Skala) der einzelnen Items überprüft. Zur Berechnung der inhaltlichen Validität der einzelnen Items (Content Validity Index [I-CVI]) wurde die Experteneinschätzung der Relevanz dichotomisiert codiert („sehr relevant“, „ziemlich relevant“ = 1; „etwas relevant“ und „gar nicht relevant“ = 0) und die Summe der Einschätzungen durch die Expertenanzahl geteilt [35]. Ein I‑CVI ≥ 0,78 gilt als exzellent [35]. Zusätzlich wurde der durchschnittliche CVI für den gesamten Fragebogen berechnet (CVI/ave). Zusätzlich konnten die Mitglieder des Expertengremiums Freitextkommentare zu jedem Item geben. Die erste Fragebogenversion wurde dann auf Basis der berechneten CVI sowie der Freitextkommentare revidiert.

### Kognitive Interviews mit Betroffenen

Im Anschluss wurden anhand der zweiten Fragebogenversion mit konsekutiv rekrutierten Patienten mit funktionellen Bewegungsstörungen sogenannte kognitive Interviews mit einer maximalen Dauer von bis zu 90 min durchgeführt, um Laienverständlichkeit, Unzweideutigkeit und Relevanz der Items aus Sicht von Betroffenen zu überprüfen. Diese Interviewmethode wird zunehmend als Mittel genutzt, um Einblick in die intrapersonell stattfindenden kognitiven Prozesse zu bekommen, die im Betroffenen beim Beantworten von Items ablaufen [1, 4]. Dabei ist von Interesse, wie Betroffene Fragen oder Begriffe verstehen, Informationen und Ereignisse aus dem Gedächtnis abrufen, Entscheidungen darüber treffen, wie sie antworten, und ihre „intern“ ermittelte Antwort formalen Antwortkategorien zuordnen. Das Ziel besteht darin, Hinweise auf problematische Formulierungen zu identifizieren [12]. Dadurch stärkt das kognitive Interview als Methode in Verbindung mit anderen Methoden (wie der strukturierten Expertenbefragung) die inhaltliche Validität von Fragebögen [4]. Entsprechend den Empfehlungen zur Durchführung kognitiver Interviews wurde eine standardisierte mit einer offenen Vorgehensweise kombiniert [39]. Zum einen wurden einige Nachfragen für jedes zu testende Item festgelegt:

- Nachfragen zum Verständnis des Items/von Begriffen: Wie haben Sie das Item „…“ verstanden? Wie haben Sie den Begriff „…“ verstanden?
- Nachfragen zum Abruf relevanter Informationen bzw. Ereignisse aus dem Gedächtnis: Wie sind Sie bei der Beantwortung des Items vorgegangen? Woran haben Sie sich erinnert?
- Nachfragen zum Entscheidungsprozess (Zuordnung der „intern“ ermittelten Antwort zu formalen Antwortkategorien): Wie kommt es, dass Sie diese Antwortkategorie gewählt haben?

Zum anderen wurden bei Bedarf spontane Nachfragen gestellt.

Es wird empfohlen, kognitive Interviews mit mindestens fünf Patienten durchzuführen [12]. Entsprechend führten wir kognitive Interviews mit sechs konsekutiv rekrutierten Patienten mit funktionellen Bewegungsstörungen durch. Die Bestimmung der letztendlichen Stichprobengröße wurde einerseits von der Bewertung der Verlässlichkeit der Antworten („confidence rating“ [CR]) der Patienten abhängig gemacht. Diese wurde von dem Interviewer auf einer Skala von 1 bis 4 eingeordnet, wobei 1 für eine hohe Verlässlichkeit steht. Andererseits wurde die Auswertung der kognitiven Interviews im Hinblick auf das Ausmaß der Notwendigkeit, Items anzupassen, um Verstehbarkeit, Eindeutigkeit und Relevanz der Items zu erhöhen, berücksichtigt.

Um einerseits systematisch vorzugehen und andererseits die Empfehlung zu berücksichtigen, dass die aufgrund der Ergebnisse der kognitiven Interviews veränderten Items einer weiteren Überprüfung unterzogen werden, kombinierten wir eine systematische mit einer iterativen Vorgehensweise, indem allen Betroffenen zunächst konsequent die zweite Fragebogenversion vorgelegt wurde und im Anschluss ihre Rückmeldung zu den Veränderungen eingeholt wurde.

Einschlägigen Empfehlungen zur Anwendung kognitiver Interviews entsprechend wurden die Patientenantworten detailliert dokumentiert und anschließend ausgewertet [12].

## Ergebnisse

### Erste Fragebogenversion

Auf der Basis der systematischen Literaturrecherche wurde eine erste Fragebogenversion mit 37 Items erstellt. Hierbei handelte es sich um sieben Fragenblöcke mit je vier bis acht Einzelitems, die auf die gleiche Stammfrage folgen: (z. B. „1. Wie oft treten folgende Probleme auf?“ – „1a: Lähmung oder Muskelschwäche“, „1b: Unkoordinierte Bewegungen“ usw.). Die auf die Stammfragen folgenden Einzelitems erfassen

- die Häufigkeit des Auftretens verschiedener Symptome (1 Fragenblock: 4 Items),
- deren Lokalisation (1 Fragenblock: 6 Items),
- die Beeinträchtigung wesentlicher Alltagsfunktionen durch die Bewegungsstörung (3 Fragenblöcke: 13 Items),
- die Beeinträchtigung der Bewegungsfähigkeit durch nichtmotorische Symptome (8 Items) und
- die Beeinträchtigung wesentlicher sozialer Bereiche durch die Bewegungsstörung (6 Items).

Die Items wurden auf einer drei- bis fünfstufigen Likert-Skala bewertet.

### Strukturierte Expertenbefragung: inhaltliche Validitätsindizes

Diese erste Fragebogenversion wurde sieben Experten (NB, GE, CG, AM, TS, AW, SP) zur strukturierten Bewertung der Verständlichkeit und der Relevanz vorgelegt. Alle Experten sind Neurologen mit einer medianen Erfahrung mit der Diagnostik und Behandlung funktioneller Bewegungsstörung von 16 Jahren (4–31 Jahre).

Unter Berücksichtigung der Expertenkommentare und -bewertungen wurden die Formulierungen von sechs Stammfragen und 14 Einzelitems verändert, vier Items wurden ergänzt, zwei Stammfragen/vier Einzelitems wurden zu einer Stammfrage/zwei Items zusammengeführt, eine Stammfrage/sechs Einzelitems wurden verschoben, zwei Items wurden gesplittet und zwei Items gelöscht (s. Tabelle S1). Alle beibehaltenen Items haben einen exzellenten I‑CVI von mindestens 0,86. Der durchschnittliche CVI-Wert des Fragebogens beträgt nach der o. g. Itemlöschung 0,97 und ist somit ebenfalls exzellent. Die zweite Version des Fragebogens besteht aus 35 Items; es handelte sich um sieben Fragenblöcke mit je drei bis sieben Einzelitems. In der ergänzenden Tabelle S3 werden die Versionen 1 und 2 gegenübergestellt und mit CVI-Werten, der detaillierten Art der Itemveränderung und den berücksichtigten Kommentaren der Experten aufgeführt.

### Kognitive Interviews

Anhand der zweiten Fragebogenversion wurden mit sechs konsekutiv rekrutierten Patienten mit funktioneller Bewegungsstörungen kognitive Interviews mit einer durchschnittlichen Dauer von 60 min durchgeführt. Die interviewte Gruppe war heterogen im Hinblick auf ihr Geschlecht (vier Frauen, zwei Männer), Alter (Median: 52 Jahre [24–81]), die Erkrankungsdauer (Median: 2 Jahre [3 Monate–11 Jahre]) sowie die Art und Schwere der funktionellen Bewegungsstörung (Bewegungsstörung mit funktionellen Myoklonien [*n* = 1], funktionelle Beinlähmung [*n* = 1], schwere funktionelle Gangstörung [*n* = 3], schwere funktionelle Hyperkinesien [*n* = 1]). Die Verlässlichkeit der Antworten war bei vier der sechs Patienten sehr gut bis gut; unter diesen Interviewten befanden sich ein Physiotherapeut und eine Medizinstudentin. In jedem Interview wurden alle Items des Fragebogens besprochen. Die Rückmeldungen der Betroffenen wurden genutzt, um sowohl die einheitliche Verständlichkeit der intendierten Bedeutung, Unzweideutigkeit und Vollständigkeit der Items aus Sicht von Betroffenen als auch die Eignung der Antwortmöglichkeiten zu überprüfen. Fast alle Teilnehmer betonten unaufgefordert, dass sie die Befragung zum Fragebogen als ein sehr positives Zeichen der Anerkennung und Sichtbarmachung ihrer ansonsten oft medizinisch vernachlässigten Beeinträchtigungen empfanden.

Unter Berücksichtigung der kognitiven Interviews wurden vier Stammfragen und zwei Einzelitems umformuliert, eine Fragebogeninstruktion sowie drei Einzelitems ergänzt, die Antwortmöglichkeiten von zwei Stammfragen von einer vier- auf eine fünfstufige Likert-Skala erweitert und bei fünf Stammfragen Unterstreichungen als stilistisches Mittel zur Verdeutlichung des Bezugsrahmens vorgenommen (s. Tabelle S2).

Die Rückmeldungen zu den bereits umgesetzten Veränderungen in den iterativ ergänzten Fragebogenversionen waren durchweg bestärkend; teilweise waren die Veränderungen direkt anschlussfähig an wiederholt auftretende Verständnisschwierigkeiten.

### Endgültige Version

**Figure 1:**
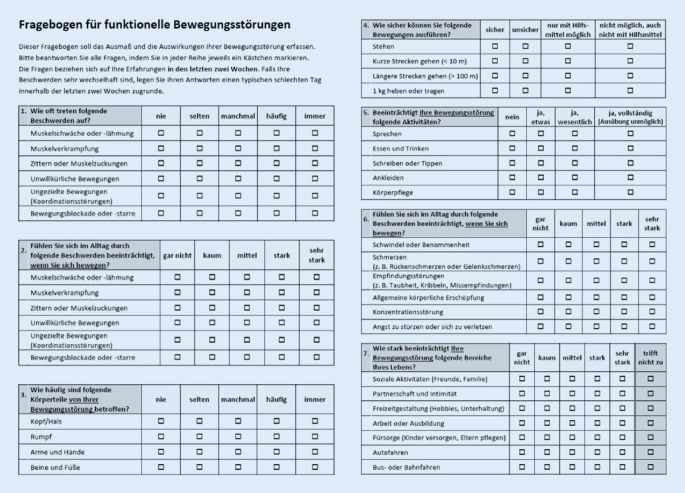

Die endgültige Version des Fragebogens besteht aus 38 Items (Abb. 1); es handelt sich um sieben Fragenblöcke mit je vier bis sieben Einzelitems. Die endgültige inhaltlich validierte Version des Fragebogens für funktionelle Bewegungsstörungen ist zusätzlich online frei als ergänzende PDF-Datei abrufbar. In der ergänzenden Tabelle S3 (online) werden die Versionen 1, 2 und 3 gegenübergestellt und detailliert die Art der Itemveränderung mit den berücksichtigten Rückmeldungen der Experten und Betroffenen aufgeführt.

## Diskussion

Funktionelle Bewegungsstörungen gelangen dank der Validierung diagnostischer Untersuchungstechniken [32] und eines integrativen biopsychosozialen Verständnisses [36] zunehmend in den Fokus wissenschaftlicher und therapeutischer Bemühungen [37]. Neben der klassischen Psychotherapie [9] werden multimodale Therapieprogramme [41] sowie innovative nichtinvasive Hirnstimulationsverfahren [42] klinisch geprüft. Um in Zukunft den Betroffenheitsgrad und einen möglichen Therapieeffekt in der klinischen Praxis und in Studien quantitativ besser erfassen zu können, entwickelten wir den ersten störungsspezifischen Fragebogen für funktionelle Bewegungsstörungen und validierten diesen inhaltlich. Neben rein motorischen Aspekten der Bewegungsstörung (semiquantitativ nach Lokalisation, Häufigkeit und Beeinträchtigung erfasst; Fragenblöcke 1–3) erfasst der Fragebogen auch die damit verbundenen Einschränkungen in den Bereichen Mobilität (Fragenblock 4, 4 Items) und Aktivitäten des täglichen Lebens (Fragenblock 5, 5 Items). Gesondert abgefragt werden zudem nichtmotorische Beschwerden, die die Mobilität und Aktivität im Alltag beeinträchtigen und besonders häufig in Verbindung mit funktionellen Bewegungsstörungen auftreten (Fragenblock 6, 6 Items). Zuletzt wird die Beeinträchtigung wesentlicher sozialer Bereiche durch die Bewegungsstörung erfasst (Fragenblock 7, 7 Items).

Funktionelle Bewegungsstörungen sind als komplexe neuropsychiatrische Störungen zu verstehen, deren Pathophysiologie Auffälligkeiten in den Domänen Aufmerksamkeit, interozeptive Wahrnehmung und multimodale sensomotorische Integration beinhaltet [43, 44]. Demnach erscheint es essenziell, das Störungsbild über die schematische Abfrage motorischer Phänomene hinaus zu erfassen. Die einzigen bislang existierenden störungsspezifischen Erhebungsinstrumente sind die durch Behandler zu bewertende PMDRS und deren Kurzversion S‑FMDRS. Die durch die S‑FMDRS quantifizierte motorische Einschränkung korreliert jedoch in einer Untersuchung von 61 Patienten nicht mit der gesundheitsbezogenen Lebensqualität; Ängste und kognitive Beschwerden hingegen waren in diesem Kollektiv statistische Prädiktoren der Lebensqualität [45]. In einer anderen Studie mit 181 Patienten mit funktionellen Bewegungsstörungen zeigte sich auch kein Zusammenhang zwischen dem Ausmaß selbstberichteter motorischer Symptomatik und der Lebensqualität, während eine signifikante inverse Korrelation mit Erschöpfung und Depressivität bestand [13]. Neben kognitiven und psychischen Beschwerden sind auch Sensibilitätsstörungen und Schwindel häufige relevante Facetten funktioneller Bewegungsstörungen [24, 40]. Somit ist davon auszugehen, dass ein Erhebungsinstrument, welches nur die motorische Phänomenologie erfasst, in keinem für Patienten relevanten Bezug zur gesundheitsbezogenen Lebensqualität steht.

Diverse relevante Aspekte einer funktionellen Bewegungsstörung im Rahmen von Studien könnten auch gesondert durch einzelne, validierte Fragebögen für Erschöpfung, Schmerzen, Angststörung usw. erhoben werden [34], jedoch würde dann der konkrete Bezug zum Bewegungsvermögen der Betroffenen fehlen. Gerade diese Beziehung zwischen nichtmotorischen Beschwerden und der Beeinträchtigung der Bewegung wurde in den kognitiven Interviews mehrfach durch Patienten angesprochen und demnach explizit in den Formulierungen mehrerer Fragen berücksichtigt. Häufig werden anstelle von störungsspezifischen Erhebungsinstrumenten allgemeinere Skalen wie die Clinical Global Impression Scale (CGI) oder Maße für gesundheitsbezogene Lebensqualität als primäre Endpunkte für Interventionsstudien angewandt [34]. Problematisch ist dabei, dass der relativ hohe Anteil an psychiatrischen und somatischen Komorbiditäten in dieser Patientengruppe relevante Therapieeffekte durch störungsspezifische Ansätze verschleiern kann. Ein störungsspezifischer Fragebogen kann hier unter Umständen eine differenziertere Abbildung der Störungsschwere und des Therapieeffekts erzielen. Schließlich ist die Anwendung mehrteiliger Testbatterien in der Routineversorgung (z. B. in der Sprechstunde oder zu verschiedenen Zeitpunkten während der stationären Rehabilitation) aufgrund des zeitlichen Aufwands der Erhebung und Auswertung weder für Betroffene noch für Behandler praktikabel.

Eine wesentliche methodische Komponente unseres Vorgehens ist die Einbeziehung von Patienten in die Fragebogenentwicklung im Rahmen der kognitiven Interviews. Dieses partizipative Vorgehen wird mittlerweile als essenzieller Bestandteil der Entwicklung von patientenzentrierten Erhebungsinstrumenten angesehen [46]. Funktionelle Bewegungsstörungen erhielten bis vor Kurzem selbst von Bewegungsstörungsspezialisten verhältnismäßig wenig Aufmerksamkeit [16]. Akademische Auseinandersetzungen zum Thema waren ebenso wie spezifische Behandlungsangebote eine Seltenheit. Seit der Jahrhundertwende ist eine erfreuliche Wiederentdeckung dieses Themas zu verzeichnen [15, 33], obwohl spezialisierte therapeutische Angebote leider weiterhin eine Ausnahme bleiben [5, 37]. Qualitative Studien zum Krankheitserleben Betroffener spiegeln dementsprechend die psychologischen Auswirkungen der geringen Beachtung wider, die diese Störungsbilder in medizinischen Strukturen erfahren [3, 23]. Daher ist es umso wichtiger, Patienten und deren subjektive Wahrnehmungen und Meinungen systematisch in die wissenschaftliche und therapeutische Entwicklung dieses klinischen Feldes einzubeziehen, insbesondere bei der Entwicklung der Endpunkte, mit denen zukünftig der Nutzen von Therapien gemessen werden soll [28].

Die vorliegende Fragebogenversion wird in einem nächsten Schritt testanalytisch in einer prospektiven, multizentrischen Studie validiert. Hierbei werden die Konstruktvalidität und Reliabilität sowie die psychometrischen Eigenschaften und die Faktorenstruktur des neuen Instruments analysiert. Im Rahmen einer Nachbefragung soll zudem die Änderungssensitivität des Instruments evaluiert werden.

## Fazit für die Praxis

Mit der Entwicklung des „Fragebogens für funktionelle Bewegungsstörungen“ liegt nun erstmals ein störungsspezifisches Erhebungsinstrument zur Patientenselbstbewertung für die klinische und wissenschaftliche Praxis vor, welches ein bislang vernachlässigtes Störungsbild in den Fokus bringt. Die klinimetrische Validierung in einer prospektiven, multizentrischen Studie ist bereits eingeleitet.

## Supplementary Information







