# Supplementary material for: Development and content validation of a questionnaire for functional movement disorders
Source: Nervenarzt. 2021 Dec 23;93(10):1009–18. [Article in German] doi: 10.1007/s00115-021-01247-1 (PMC9534974; doi:10.1007/s00115-021-01247-1)
Supplement: Supplementary file 1 [file 115_2021_1247_MOESM1_ESM.pdf]

Tabelle S1: Veränderungen entsprechend den Expertenkommentare und -bewertungen

Art der Veränderung	Gegenstand der Veränderung	Beispiel
Umformulierung	6 Stammfragen und 14 Einzelitems	Stammfrage: „Wie stark beeinträchtigt Ihre Bewegungsstörung folgende Bereiche Ihres Lebens?“ statt „Wie stark wirkt sich Ihre Bewegungsstörung auf folgende Bereiche des Lebens aus?“; Einzelitem: „Muskelschwäche oder –lähmung“ statt „Lähmung oder Muskelschwäche“.
Zusammenlegung	2 Stammfragen	Die Stammfrage „Wie sicher können Sie folgende Bewegungen ohne Unterstützung oder Hilfsmittel ausführen?“ wurde mit „Wie sicher können Sie folgende Bewegungen mit Unterstützung oder Hilfsmittel ausführen?“ zu der Stammfrage „Wie sicher können Sie folgende Bewegungen ausführen?“ mit Antwortmöglichkeiten auf einer vierstufigen Likert-Skala von „sicher“ bis „nicht möglich, auch nicht mit Hilfsmittel“ vereint.
	4 Einzelitems	Die Einzelitems „Linkes Bein“ und „Rechtes Bein“ wurden zu dem Einzelitem „Beine und Füße“ verschmolzen.
Verschiebung	1 Stammfrage	Die Stammfrage „Fühlen Sie sich durch folgende Beschwerden beeinträchtigt, wenn Sie sich bewegen?“ wurde direkt an den

		Fragenblock Wie oft treten folgende Beschwerden auf? angeschlossen.
	6 Einzelitems	In dem Fragenblock „Beeinträchtigt Ihre Bewegungsstörung folgende Aktivitäten?“ wurde das Einzelitem „Essen und Trinken“ vor das Item „Schreiben und Tippen“ gezogen.
Ergänzung	4 Einzelitems	Das Einzelitem „Muskelverkrampfung“ wurde in dem Fragenblock „Fühlen Sie sich durch folgende Beschwerden beeinträchtigt, wenn Sie sich bewegen?“ ergänzt, um alle Beschwerden des vorherigen Fragenblocks „Wie oft treten folgende Probleme auf?“ aufzugreifen.
Splitting	2 Einzelitems	Das Item „Unkoordinierte Bewegungen“ wurde in das Item „Unwillkürliche Bewegungen“ und das Item „Ungezielte Bewegungen (Koordinationsstörungen)“ geteilt.
Löschung	2 Einzelitems	Einzelitems, die sich auf die Fähigkeit „Tasche tragen“ bezogen, wurden gelöscht.
