# Supplementary material for: Development and content validation of a questionnaire for functional movement disorders
Source: Nervenarzt. 2021 Dec 23;93(10):1009–18. [Article in German] doi: 10.1007/s00115-021-01247-1 (PMC9534974; doi:10.1007/s00115-021-01247-1)
Supplement: Supplementary file 2 [file 115_2021_1247_MOESM2_ESM.pdf]

Tabelle S2: Veränderungen entsprechend den kognitiven Interviews mit Betroffenen

Problemfeld	Art der Veränderung	Gegenstand der Veränderung	Beispiele
Undeutlicher Bezugsrahmen	Unterstreichung	4 Stammfragen	„Beeinträchtigt Ihre Bewegungsstörung folgende Aktivitäten?" oder „Fühlen Sie sich durch folgende Beschwerden beeinträchtigt, wenn Sie sich bewegen?"
	Umformulierung	3 Stammfragen; 1 Einzelitem	„Fühlen Sie sich *im Alltag* durch folgende Beschwerden beeinträchtigt, wenn Sie sich bewegen?" statt „Fühlen Sie sich durch folgende Beschwerden beeinträchtigt, wenn Sie sich bewegen?";<br>„Wie oft treten folgende Beschwerden auf?" statt „Wie oft treten folgende Probleme auf?"
	Umformulierung und Unterstreichung	1 Stammfrage	„Wie häufig sind folgende Körperteile von Ihrer Bewegungsstörung betroffen?" statt „Wie häufig sind folgende Körperteile betroffen?"
	Ergänzung	Fragebogeninstruktion	Falls Ihre Beschwerden sehr wechselhaft sind, legen Sie Ihren Antworten einen typischen schlechten Tag innerhalb der letzten zwei Wochen zugrunde.

Fehlender Aspekt	Ergänzung	3 Einzelitems	Das Einzelitem „Bewegungsblockade oder –starre“ wurde als Antwortmöglichkeit auf die Stammfragen „Wie oft treten folgende Beschwerden auf?“ und „Fühlen Sie sich im Alltag durch folgende Beschwerden beeinträchtigt, wenn Sie sich bewegen?“ ergänzt; das Einzelitems „1 kg heben oder tragen“ wurde als Antwortmöglichkeit auf die Stammfrage „Wie sicher können Sie folgende Bewegungen ausführen?“ ergänzt
Fehlende Möglichkeit zur Differenzierung	Ergänzung	2 Fragenblöcke	Die vierstufige Likert-Skala (1: „gar nicht“ bis 4: „stark“) wurde auf eine fünfstufige Likert-Skala (1: „gar nicht“ bis 5: „sehr stark“) erweitert
Missverständliche Formulierung	Umformulierung	1 Einzelitem	„Stehen“ statt „Frei stehen“ als zu beantwortendes Item auf die Frage „Wie sicher können Sie folgende Bewegungen ausführen?“, da sonst die Antwortmöglichkeiten „nur mit Hilfsmittel möglich“ und „nicht möglich, auch nicht mit Hilfsmittel“ nicht passen.
