# Supplementary material for: Development and content validation of a questionnaire for functional movement disorders
Source: Nervenarzt. 2021 Dec 23;93(10):1009–18. [Article in German] doi: 10.1007/s00115-021-01247-1 (PMC9534974; doi:10.1007/s00115-021-01247-1)
Supplement: Supplementary file 3 [file 115_2021_1247_MOESM3_ESM.pdf]

Tabelle S3: Detaillierte Aufstellung der Veränderungen

CVI: Content Validity Index; KI: Kognitive Interviews; die Veränderungen sind jeweils gelb markiert

Fragebogen für funktionelle Bewegungsstörungen

Dieser Fragebogen soll das Ausmaß und die Auswirkungen Ihrer Bewegungsstörung erfassen.
Bitte beantworten Sie alle Fragen, indem Sie in jeder Reihe jeweils ein Kästchen markieren.
Die Fragen beziehen sich auf Ihre Erfahrungen **in den letzten zwei Wochen**.
Falls Ihre Beschwerden sehr wechselhaft sind, legen Sie Ihren Antworten einen typischen schlechten Tag innerhalb der letzten zwei Wochen zugrunde.

	**KI:**<br>**Umformulierung (Fragebogeninstruktion)**<br>• Konkretisierung der Anweisung, um Betroffenen mit stark fluktuierenden Beschwerden das Ausfüllen zu erleichtern.

Version 1	Version 2	Version 3
**1a. Version 1: Wie oft treten folgende Probleme auf?**	**1a. Version 2: Wie oft treten folgende Probleme auf?**	**1a. Version 3: Wie oft treten folgende Beschwerden auf?**
Lähmung oder Muskelschwäche	Muskelschwäche oder –lähmung	Muskelschwäche oder –lähmung
**nie | selten | manchmal | häufig | immer**	**nie | selten | manchmal | häufig | immer**	**nie | selten | manchmal | häufig | immer**
☐ ☐ ☐ ☐ ☐	☐ ☐ ☐ ☐ ☐	☐ ☐ ☐ ☐ ☐

**CVI :** 1,0<br>**Umformulierung (Einzelitem)**<br>Berücksichtigter Kommentar:<br>• *„Vielleicht wäre Muskellähmung besser?“*	**KI:**<br>**Umformulierung (Stammfrage)**<br>• Konkordante Begrifflichkeit unterstützt Betroffene, Fragenblock 1 und 2 miteinander in Zusammenhang zu bringen.

Version 1	Version 2	Version 3
**1c. Version 1: Wie oft treten folgende Probleme auf?**	**1b. Version 2: Wie oft treten folgende Probleme auf?**	**1b. Version 3: Wie oft treten folgende Beschwerden auf?**
Verkrampfung	Muskelverkrampfung	Muskelverkrampfung
**nie | selten | manchmal | häufig | immer**	**nie | selten | manchmal | häufig | immer**	**nie | selten | manchmal | häufig | immer**
☐ ☐ ☐ ☐ ☐	☐ ☐ ☐ ☐ ☐	☐ ☐ ☐ ☐ ☐

**CVI :** 1,0<br>**Umformulierung (Einzelitem)**<br>Berücksichtigter Kommentar:<br>• *„Muskelverkrampfung fände ich besser.“*<br>**Itemverschiebung innerhalb des Fragenblocks**<br>Berücksichtigter Kommentar:<br>• *„Aus meiner Sicht besser: Reihenfolge der Fragen 1a-d ändern.“*	**KI:**<br>**Umformulierung (Stammfrage)**<br>• Konkordante Begrifflichkeit unterstützt Betroffene, Fragenblock 1 und 2 miteinander in Zusammenhang zu bringen.

1d.	**Version 1:** **Wie oft treten folgende Probleme auf?**			
	Zittern oder Zucken			
**nie**	**selten**	**manchmal**	**häufig**	**immer**
☐	☐	☐	☐	☐

1c.	**Version 2:** **Wie oft treten folgende Probleme auf?**			
	Zittern oder Muskelzuckungen			
**nie**	**selten**	**manchmal**	**häufig**	**immer**
☐	☐	☐	☐	☐

1c.	**Version 3:** **Wie oft treten folgende Beschwerden auf?**			
	Zittern oder Muskelzuckungen			
**nie**	**selten**	**manchmal**	**häufig**	**immer**
☐	☐	☐	☐	☐

<u>**CVI :**</u> **1,0**
Umformulierung (Einzelitem)
Berücksichtigter Kommentar:

- *„Aus meiner Sicht besser: Zittern oder Muskelzuckungen.“*

Itemverschiebung innerhalb des Fragenblocks
Berücksichtigter Kommentar:

- *„Aus meiner Sicht besser: Reihenfolge der Fragen 1a-d ändern.“*

<u>**KI:**</u>
Umformulierung (Stammfrage)

- Konkordante Begrifflichkeit unterstützt Betroffene, Fragenblock 1 und 2 miteinander in Zusammenhang zu bringen.

1b.	**Version 1:** **Wie oft treten folgende Probleme auf?**			
	Unkoordinierte Bewegungen			
**nie**	**selten**	**manchmal**	**häufig**	**immer**
☐	☐	☐	☐	☐

1d.	**Version 2:** **Wie oft treten folgende Probleme auf?**			
	Unwillkürliche Bewegungen			
**nie**	**selten**	**manchmal**	**häufig**	**immer**
☐	☐	☐	☐	☐

1d.	**Version 3:** **Wie oft treten folgende Beschwerden auf?**			
	Unwillkürliche Bewegungen			
**nie**	**selten**	**manchmal**	**häufig**	**immer**
☐	☐	☐	☐	☐

<u>**CVI :**</u> **1,0**
Umformulierung (Einzelitem) und Itemsplitting
Berücksichtigte Kommentare:

<u>**KI:**</u>
Umformulierung (Stammfrage)

• *„Ich würde hier zwischen unwillkürlichen Bewegungen und Koordinationsstörungen unterscheiden (2 items).“*<br>• *„Unkoordiniert kann auch als unwillkürlich mißverstanden werden.“*<br>**Itemverschiebung innerhalb des Fragenblocks**<br>Berücksichtigter Kommentar:<br>• *„Aus meiner Sicht besser: Reihenfolge der Fragen 1a-d ändern.“*	• Konkordante Begrifflichkeit unterstützt Betroffene, Fragenblock 1 und 2 miteinander in Zusammenhang zu bringen.

1b. Version 1: Wie oft treten folgende Probleme auf?

Unkoordinierte Bewegungen

nie	selten	manchmal	häufig	immer
☐	☐	☐	☐	☐

1e. Version 2: Wie oft treten folgende Probleme auf?

Ungezielte Bewegungen (Koordinationsstörungen)

nie	selten	manchmal	häufig	immer
☐	☐	☐	☐	☐

1e. Version 3: Wie oft treten folgende Beschwerden auf?

Ungezielte Bewegungen (Koordinationsstörungen)

nie	selten	manchmal	häufig	immer
☐	☐	☐	☐	☐

**CVI : 1,0**<br>**Umformulierung (Einzelitem) und Itemsplitting**<br>Berücksichtigte Kommentare:<br>• *„Ich würde hier zwischen unwillkürlichen Bewegungen und Koordinationsstörungen unterscheiden (2 items).“*<br>• *„Aus meiner Sicht verständlicher: Ungezielte Bewegungen.“*<br>**Itemverschiebung innerhalb des Fragenblocks**<br>Berücksichtigter Kommentar:<br>• *„Aus meiner Sicht besser: Reihenfolge der Fragen 1a-d ändern.“*	**KI:**<br>**Umformulierung (Stammfrage)**<br>• Konkordante Begrifflichkeit unterstützt Betroffene, Fragenblock 1 und 2 miteinander in Zusammenhang zu bringen.

		**1f. Version 3: Wie oft treten folgende Beschwerden auf?**<br>Bewegungsblockade oder -starre<br>**nie** ☐ **selten** ☐ **manchmal** ☐ **häufig** ☐ **immer** ☐

	**<u>KI:</u>**<br>**Ergänzung (Einzelitem)**<br>• Ergänzte Beschwerde im Rahmen einer funktionellen Bewegungsstörung

**6e. Version 1: Wie wirken sich folgende Probleme auf Ihre Bewegungsfähigkeit aus?**<br>Minderbeweglichkeit (z. B. Schwäche oder Ermüdbarkeit der Muskeln)<br>**Kaum** ☐ **mittel** ☐ **stark** ☐	**2a. Version 2: Fühlen Sie sich durch folgende Beschwerden beeinträchtigt, wenn Sie sich bewegen?**<br>Muskelschwäche oder -lähmung<br>**gar nicht** ☐ **kaum** ☐ **mittel** ☐ **stark** ☐	**2a. Version 3: Fühlen Sie sich im Alltag durch folgende Beschwerden beeinträchtigt, <u>wenn Sie sich bewegen</u>?**<br>Muskelschwäche oder -lähmung<br>**gar nicht** ☐ **kaum** ☐ **mittel** ☐ **stark** ☐ **sehr stark** ☐

**<u>CVI :</u> 1,0**<br>**Umformulierung (Stammfrage)**<br>Berücksichtigte Kommentare:<br>• *„Aus meiner Sicht klarerer Info: Fühlen sie sich durch folgende Beschwerden beeinträchtigt: Gar nicht, kaum, mittel, stark.“*<br>• *„Hier sollte das Wording mit Sektion 1 konkordant sein (Also Lähmungen, Unwillkürliche Bewegungen, Koordinationsstörungen).“*<br>**Verschiebung der Stammfrage innerhalb des Fragebogens**<br>Berücksichtigter Kommentar:	**<u>KI:</u>**<br>**Umformulierung und Unterstreichung (Stammfrage)**<br>• Ergänzung „im Alltag“ verdeutlicht den Kontext, in dem die Beeinträchtigung durch die Bewegungsstörung beurteilt werden soll.<br>• Unterstreichung unterstützt Betroffene, die Beeinträchtigung konsequent auf ihre Bewegungsfähigkeit zu beziehen.<br>**Ergänzung (Antwortmöglichkeit)**<br>• Ergänzung „sehr stark“ ermöglicht den Betroffenen eine weitere Differenzierung zwischen „stark“ und „sehr stark“.

• *„Man könnte die Punkte die unter 6 stehen als eine Ergänzung der Punkte 1a-d für den Schweregrad ansehen.“*	

	**2b. Version 2: Fühlen Sie sich durch folgende Beschwerden beeinträchtigt, wenn Sie sich bewegen?**<br>Muskelverkrampfung<br><br>**gar nicht** ☐ | **kaum** ☐ | **mittel** ☐ | **stark** ☐	**2b. Version 3: Fühlen Sie sich im Alltag durch folgende Beschwerden beeinträchtigt, <u>wenn Sie sich bewegen</u>?**<br>Muskelverkrampfung<br><br>**gar nicht** ☐ | **kaum** ☐ | **mittel** ☐ | **stark** ☐ | **sehr stark** ☐

**Ergänzung (Einzelitem)**<br>Berücksichtigter Kommentar:<br>• *„Man könnte die Punkte die unter 6 stehen als eine Ergänzung der Punkte 1a-d für den Schweregrad ansehen.“*	**<u>KI:</u>**<br>**Umformulierung und Unterstreichung (Stammfrage)**<br>• Ergänzung „im Alltag“ verdeutlicht den Kontext, in dem die Beeinträchtigung durch die Bewegungsstörung beurteilt werden soll.<br>• Unterstreichung unterstützt Betroffene, die Beeinträchtigung konsequent auf ihre Bewegungsfähigkeit zu beziehen.<br>**Ergänzung (Antwortmöglichkeit)**<br>• Ergänzung „sehr stark“ ermöglicht den Betroffenen eine weitere Differenzierung zwischen „stark“ und „sehr stark“.

	**2c. Version 2: Fühlen Sie sich durch folgende Beschwerden beeinträchtigt, wenn Sie sich bewegen?**<br>Zittern oder Muskelzuckungen<br><br>**gar nicht** ☐ | **kaum** ☐ | **mittel** ☐ | **stark** ☐	**2c. Version 3: Fühlen Sie sich im Alltag durch folgende Beschwerden beeinträchtigt, <u>wenn Sie sich bewegen</u>?**<br>Zittern oder Muskelzuckungen<br><br>**gar nicht** ☐ | **kaum** ☐ | **mittel** ☐ | **stark** ☐ | **sehr stark** ☐

Ergänzung (Einzelitem)	KI:
**Ergänzung (Einzelitem)**<br>Berücksichtigter Kommentar:<br>• *„Man könnte die Punkte die unter 6 stehen als eine Ergänzung der Punkte 1a-d für den Schweregrad ansehen."*	**KI:**<br>**Umformulierung und Unterstreichung (Stammfrage)**<br>• Ergänzung „im Alltag" verdeutlicht den Kontext, in dem die Beeinträchtigung durch die Bewegungsstörung beurteilt werden soll.<br>• Unterstreichung unterstützt Betroffene, die Beeinträchtigung konsequent auf ihre Bewegungsfähigkeit zu beziehen.<br>**Ergänzung (Antwortmöglichkeit)**<br>• Ergänzung „sehr stark" ermöglicht den Betroffenen eine weitere Differenzierung zwischen „stark" und „sehr stark".

Version 1:

6d.	Wie wirken sich folgende Probleme auf Ihre Bewegungsfähigkeit aus?		
	Unwillkürliche Bewegungen (z. B. Zittern oder Zuckungen)		
	kaum	mittel	stark
	☐	☐	☐

Version 2:

2d.	Fühlen Sie sich durch folgende Beschwerden beeinträchtigt, wenn Sie sich bewegen?			
	Unwillkürliche Bewegungen			
gar nicht	kaum	mittel	stark	
☐	☐	☐	☐	

Version 3:

2d.	Fühlen Sie sich im Alltag durch folgende Beschwerden beeinträchtigt, <u>wenn Sie sich bewegen</u>?			
	Unwillkürliche Bewegungen			
gar nicht	kaum	mittel	stark	sehr stark
☐	☐	☐	☐	☐

CVI : 1,0	KI:
**CVI : 1,0**<br>**Umformulierung (Stammfrage und Einzelitem)**<br>Berücksichtigte Kommentare:<br>• *„Aus meiner Sicht klarerer Info: Fühlen sie sich durch folgende Beschwerden beeinträchtigt: Gar nicht, kaum, mittel, stark."*<br>• *„Hier sollte das Wording mit Sektion 1 konkordant sein (Also Lähmungen, Unwillkürliche Bewegungen,…)."*<br>**Verschiebung der Stammfrage innerhalb des Fragebogens**<br>Berücksichtigter Kommentar:<br>• *„Man könnte die Punkte die unter 6 stehen als eine Ergänzung der Punkte 1a-d für den Schweregrad ansehen."*	**KI:**<br>**Umformulierung und Unterstreichung (Stammfrage)**<br>• Ergänzung „im Alltag" verdeutlicht den Kontext, in dem die Beeinträchtigung durch die Bewegungsstörung beurteilt werden soll.<br>• Unterstreichung unterstützt Betroffene, die Beeinträchtigung konsequent auf ihre Bewegungsfähigkeit zu beziehen.<br>**Ergänzung (Antwortmöglichkeit)**<br>• Ergänzung „sehr stark" ermöglicht den Betroffenen eine weitere Differenzierung zwischen „stark" und „sehr stark".

	Version 2	Version 3
	**2e.** **Version 2: Fühlen Sie sich durch folgende Beschwerden beeinträchtigt, wenn Sie sich bewegen?**<br>Ungezielte Bewegungen (Koordinationsstörungen)<br>**gar nicht** ☐ | **kaum** ☐ | **mittel** ☐ | **stark** ☐	2e. **Version 3: Fühlen Sie sich im Alltag durch folgende Beschwerden beeinträchtigt, wenn Sie sich bewegen?**<br>Ungezielte Bewegungen (Koordinationsstörungen)<br>**gar nicht** ☐ | **kaum** ☐ | **mittel** ☐ | **stark** ☐ | **sehr stark** ☐

**Ergänzung (Einzelitem)**	**KI:**
Berücksichtigter Kommentar:<br>• *„Man könnte die Punkte die unter 6 stehen als eine Ergänzung der Punkte 1a-d für den Schweregrad ansehen.“*	**Umformulierung und Unterstreichung (Stammfrage)**<br>• Ergänzung „im Alltag“ verdeutlicht den Kontext, in dem die Beeinträchtigung durch die Bewegungsstörung beurteilt werden soll.<br>• Unterstreichung unterstützt Betroffene, die Beeinträchtigung konsequent auf ihre Bewegungsfähigkeit zu beziehen.<br>**Ergänzung (Antwortmöglichkeit)**<br>• Ergänzung „sehr stark“ ermöglicht den Betroffenen eine weitere Differenzierung zwischen „stark“ und „sehr stark“.

		**2f.** **Version 3: Fühlen Sie sich im Alltag durch folgende Beschwerden beeinträchtigt, wenn Sie sich bewegen?**<br>Bewegungsblockade oder -starre<br>**nie** ☐ | **selten** ☐ | **manchmal** ☐ | **häufig** ☐ | **immer** ☐

	**KI:**<br>**Ergänzung (Einzelitem)**<br>• Ergänzte Beschwerde im Rahmen einer funktionellen Bewegungsstörung

	Version 1:			
**2a.**	**Welche Körperteile sind betroffen?**			
	Kopf/Gesicht			
**nie**	**selten**	**manchmal**	**häufig**	**immer**
☐	☐	☐	☐	☐

	Version 2:			
**3a.**	**Wie häufig sind folgende Körperteile betroffen?**			
	Kopf/Hals			
**nie**	**selten**	**manchmal**	**häufig**	**immer**
☐	☐	☐	☐	☐

	Version 3:			
**3a.**	**Wie häufig sind folgende Körperteile von Ihrer Bewegungsstörung betroffen?**			
	Kopf/Hals			
**nie**	**selten**	**manchmal**	**häufig**	**immer**
☐	☐	☐	☐	☐

**CVI : 0.86**<br>**Umformulierung (Einzelitem und Stammfrage)**<br>Berücksichtigte Kommentare:<br>• *„Die Frage ist verständlich aber passt nicht so ganz zu den Antwortmöglichkeiten. Wie häufig sind diese Körperteile betroffen?“*<br>• *„Sollte hier oder unter Punkt 2b auch der Hals und Nacken mit aufgenommen werden?“*	**KI:**<br>**Ergänzung (Stammfrage)**<br>• Ergänzung „von Ihrer Bewegungsstörung“ verdeutlicht, dass die Betroffenheit durch die Bewegungsstörung und nicht durch andere, z.B. sensible Symptome (wie Kribbeln, Schmerzen, etc.) beurteilt werden soll.

	Version 1:			
**2b.**	**Welche Körperteile sind betroffen?**			
	Rumpf			
**nie**	**selten**	**manchmal**	**häufig**	**immer**
☐	☐	☐	☐	☐

	Version 2:			
**3b.**	**Wie häufig sind folgende Körperteile betroffen?**			
	Rumpf			
**nie**	**selten**	**manchmal**	**häufig**	**immer**
☐	☐	☐	☐	☐

	Version 3:			
**3b.**	**Wie häufig sind folgende Körperteile von Ihrer Bewegungsstörung betroffen?**			
	Rumpf			
**nie**	**selten**	**manchmal**	**häufig**	**immer**
☐	☐	☐	☐	☐

CVI : 0.86	KI:
**Umformulierung (Stammfrage)**<br>Berücksichtigter Kommentar:<br>• *„Die Frage ist verständlich aber passt nicht so ganz zu den Antwortmöglichkeiten. Wie häufig sind diese Körperteile betroffen?“*	**Ergänzung (Stammfrage)**<br>• Ergänzung „von Ihrer Bewegungsstörung“ verdeutlicht, dass die Betroffenheit durch die Bewegungsstörung und nicht durch andere, z.B. sensible Symptome (wie Kribbeln, Schmerzen, etc.) beurteilt werden soll.

	Version 1:			
2c.	**Welche Körperteile sind betroffen?**			
	Linker Arm			
**nie**	**selten**	**manchmal**	**häufig**	**immer**
☐	☐	☐	☐	☐

	Version 2:			
3c.	**Wie häufig sind folgende Körperteile betroffen?**			
	Arme und Hände			
**nie**	**selten**	**manchmal**	**häufig**	**immer**
☐	☐	☐	☐	☐

	Version 3:			
3c.	**Wie häufig sind folgende Körperteile von Ihrer Bewegungsstörung betroffen?**			
	Arme und Hände			
**nie**	**selten**	**manchmal**	**häufig**	**immer**
☐	☐	☐	☐	☐

CVI : 0.86	KI:
**Umformulierung (Einzelitem und Stammfrage)**<br>Berücksichtigte Kommentare:<br>• *„Die Frage ist verständlich aber passt nicht so ganz zu den Antwortmöglichkeiten. Wie häufig sind diese Körperteile betroffen?“*<br>• *„Sollte unter Punkt 2c und d die Hand und unter Punkt 2e und f der Fuß mit aufgenommen werden?“*<br>**Zusammenlegung zweier Einzelitems**<br>Berücksichtigter Kommentar:<br>• *„Aber warum li/re Arm? Was ist die Idee dahinter?“*	**Ergänzung (Stammfrage)**<br>• Ergänzung „von Ihrer Bewegungsstörung“ verdeutlicht, dass die Betroffenheit durch die Bewegungsstörung und nicht durch andere, z.B. sensible Symptome (wie Kribbeln, Schmerzen, etc.) beurteilt werden soll.

	Version 1:
2d.	**Welche Körperteile sind betroffen?**
	Linkes Bein

	Version 2:
3d.	**Wie häufig sind folgende Körperteile betroffen?**
	Beine und Füße

	Version 3:
3d.	**Wie häufig sind folgende Körperteile von Ihrer Bewegungsstörung betroffen?**
	Beine und Füße

nie	selten	manchmal	häufig	immer
☐	☐	☐	☐	☐

nie	selten	manchmal	häufig	immer
☐	☐	☐	☐	☐

nie	selten	manchmal	häufig	immer
☐	☐	☐	☐	☐

CVI : 0.86
Umformulierung (Einzelitem und Stammfrage)
Berücksichtigte Kommentare:

- *„Die Frage ist verständlich aber passt nicht so ganz zu den Antwortmöglichkeiten. Wie häufig sind diese Körperteile betroffen?"*
- *„Sollte unter Punkt 2c und d die Hand und unter Punkt 2e und f der Fuß mit aufgenommen werden?"*

Zusammenlegung zweier Einzelitems
Berücksichtigter Kommentar:

- *„Aber warum li/re Arm? Was ist die Idee dahinter?"*

KI:
Ergänzung (Stammfrage)

- Ergänzung „von Ihrer Bewegungsstörung" verdeutlicht, dass die Betroffenheit durch die Bewegungsstörung und nicht durch andere, z.B. sensible Symptome (wie Kribbeln, Schmerzen, etc.) beurteilt werden soll.

3a. Version 1:
Wie sicher können Sie folgende Bewegungen ohne Unterstützung oder Hilfsmittel ausführen?

Frei stehen

sicher	unsicher	unmöglich
☐	☐	☐

4a. Version 2:

Wie sicher können Sie folgende Bewegungen ausführen?

Frei stehen

sicher	unsicher	nur mit Hilfsmittel möglich	nicht möglich, auch nicht mit Hilfsmittel
☐	☐	☐	☐

4a. Version 3:

Wie sicher können Sie folgende Bewegungen ausführen?

Stehen

sicher	unsicher	nur mit Hilfsmittel möglich	nicht möglich, auch nicht mit Hilfsmittel
☐	☐	☐	☐

CVI: 1,0
Umformulierung und Zusammenlegung zweier Stammfragen
Berücksichtigter Kommentar:

- *„Alternativ 3 und 4 zusammenfassen: Sicher, unsicher, nur mit Hilfsmittel, unmöglich."*

KI:
Umformulierung

- Streichung von „frei", da sonst die dritte und vierte Antwortmöglichkeit nicht passen.

3b.	Version 1:<br>**Wie sicher können Sie folgende Bewegungen ohne Unterstützung oder Hilfsmittel ausführen?**	
	Kurze Strecken gehen	
**sicher**	**unsicher**	**unmöglich**
☐	☐	☐

4b.	Version 2:<br><br>**Wie sicher können Sie folgende Bewegungen ausführen?**		
	Kurze Strecken gehen (< 10 m)		
**sicher**	**unsicher**	**nur mit Hilfsmittel möglich**	**nicht möglich, auch nicht mit Hilfsmittel**
☐	☐	☐	☐

4b.	Version 3:<br><br>**Wie sicher können Sie folgende Bewegungen ausführen?**		
	Kurze Strecken gehen (< 10 m)		
**sicher**	**unsicher**	**nur mit Hilfsmittel möglich**	**nicht möglich, auch nicht mit Hilfsmittel**
☐	☐	☐	☐

**CVI: 1,0**<br>**Umformulierung (Einzelitem und Stammfrage) und Zusammenlegung zweier Stammfragen**<br>Berücksichtigte Kommentare:<br>• *„Alternativ 3 und 4 zusammenfassen: Sicher, unsicher, nur mit Hilfsmittel, unmöglich."*<br>• *„Eine Entfernungsangabe (10m ?) wäre hilfreich."*	**KI:**<br>• Keine Veränderung

3c.	Version 1:<br>**Wie sicher können Sie folgende Bewegungen ohne Unterstützung oder Hilfsmittel ausführen?**	
	Längere Strecken gehen	
**sicher**	**unsicher**	**unmöglich**
☐	☐	☐

4c.	Version 2:<br><br>**Wie sicher können Sie folgende Bewegungen ausführen?**		
	Längere Strecken gehen (> 100 m)		
**sicher**	**unsicher**	**nur mit Hilfsmittel möglich**	**nicht möglich, auch nicht mit Hilfsmittel**
☐	☐	☐	☐

4c.	Version 3:<br><br>**Wie sicher können Sie folgende Bewegungen ausführen?**		
	Längere Strecken gehen (> 100 m)		
**sicher**	**unsicher**	**nur mit Hilfsmittel möglich**	**nicht möglich, auch nicht mit Hilfsmittel**
☐	☐	☐	☐

**CVI: 1,0**<br>**Umformulierung (Einzelitem und Stammfrage) und Zusammenlegung zweier Stammfragen**<br>Berücksichtigte Kommentare:<br>• *„Alternativ 3 und 4 zusammenfassen: Sicher, unsicher, nur mit Hilfsmittel, unmöglich."*<br>• *„Eine Entfernungsangabe wäre hilfreich."*	**KI:**<br>• Keine Veränderung

4d. **Version 3:**
Wie sicher können Sie folgende Bewegungen ausführen?

1 kg heben oder tragen

sicher	unsicher	nur mit Hilfsmittel möglich	nicht möglich, auch nicht mit Hilfsmittel
☐	☐	☐	☐

	**KI:**<br>**Ergänzung (Einzelitem)**<br>• Ergänzung der Belastbarkeit der oberen Extremitäten.

5a. Version 1: Wie wirkt sich Ihre Bewegungsstörung auf folgende Alltagsaktivitäten aus?			
Sprechen			
normal	etwas eingeschränkt	wesentlich eingeschränkt	unmöglich
☐	☐	☐	☐

5a. Version 2: **Beeinträchtigt Ihre Bewegungsstörung folgende Aktivitäten?**			
Sprechen			
nein	ja, etwas	ja, wesentlich	ja, vollständig (Ausübung unmöglich)
☐	☐	☐	☐

5a. Version 2: Beeinträchtigt Ihre Bewegungsstörung folgende Aktivitäten?			
Sprechen			
nein	ja, etwas	ja, wesentlich	ja, vollständig (Ausübung unmöglich)
☐	☐	☐	☐

CVI : 0,86	KI:
**Umformulierung (Stammfrage)**<br>Berücksichtigter Kommentar:<br>• *„Aus meiner Sicht verständlicher: Beeinträchtigt ihre Bewegungsstörung …? Nein, etwas, wesentlich,…“*	**Unterstreichung (Stammfrage)**<br>• Unterstreichung unterstützt Betroffene, die Beeinträchtigung konsequent auf die Bewegungsstörung zu beziehen.

5c. Version 1: Wie wirkt sich Ihre Bewegungsstörung auf folgende Alltagsaktivitäten aus?			
Essen und Trinken			
normal	etwas eingeschränkt	wesentlich eingeschränkt	unmöglich
☐	☐	☐	☐

5b. Version 2: **Beeinträchtigt Ihre Bewegungsstörung folgende Aktivitäten?**			
Essen und Trinken			
nein	ja, etwas	ja, wesentlich	ja, vollständig (Ausübung unmöglich)
☐	☐	☐	☐

5b. Version 2: Beeinträchtigt Ihre Bewegungsstörung folgende Aktivitäten?			
Essen und Trinken			
nein	ja, etwas	ja, wesentlich	ja, vollständig (Ausübung unmöglich)
☐	☐	☐	☐

CVI : 0,86	KI:
**Umformulierung (Stammfrage)**<br>Berücksichtigter Kommentar:<br>• *„Aus meiner Sicht verständlicher: Beeinträchtigt ihre Bewegungsstörung …? Nein, etwas, wesentlich,…“*<br>**Itemverschiebung innerhalb Fragenblock**<br>Berücksichtigter Kommentar:<br>• *„andere Reihenfolge perhaps? Sprechen – Essen/Trinken – Schreiben.“*	**Unterstreichung (Stammfrage)**<br>• Unterstreichung unterstützt Betroffene, die Beeinträchtigung konsequent auf die Bewegungsstörung zu beziehen.

5b. Version 1:
Wie wirkt sich Ihre Bewegungsstörung auf folgende Alltagsaktivitäten aus?

Schreiben oder Tippen

normal	etwas eingeschränkt	wesentlich eingeschränkt	unmöglich
☐	☐	☐	☐

5c. Version 2:
Beeinträchtigt Ihre Bewegungsstörung folgende Aktivitäten?

Schreiben oder Tippen

nein	ja, etwas	ja, wesentlich	ja, vollständig (Ausübung unmöglich)
☐	☐	☐	☐

5c. Version 2:
Beeinträchtigt Ihre Bewegungsstörung folgende Aktivitäten?

Schreiben oder Tippen

nein	ja, etwas	ja, wesentlich	ja, vollständig (Ausübung unmöglich)
☐	☐	☐	☐

CVI : 1,0	KI:
**Umformulierung (Stammfrage)**<br>Berücksichtigter Kommentar:<br>• *„Aus meiner Sicht verständlicher: Beeinträchtigt ihre Bewegungsstörung …? Nein, etwas, wesentlich,…“*<br>**Itemverschiebung innerhalb des Fragenblocks**<br>Berücksichtigter Kommentar:<br>• *„andere Reihenfolge perhaps? Sprechen – Essen/Trinken – Schreiben.“*	**Unterstreichung (Stammfrage)**<br>• Unterstreichung unterstützt Betroffene, die Beeinträchtigung konsequent auf die Bewegungsstörung zu beziehen.

5d.	Version 1: Wie wirkt sich Ihre Bewegungsstörung auf folgende Alltagsaktivitäten aus?		
Ankleiden			
normal	etwas eingeschränkt	wesentlich eingeschränkt	unmöglich
☐	☐	☐	☐

5d.	Version 2: Beeinträchtigt Ihre Bewegungsstörung folgende Aktivitäten?		
Ankleiden			
nein	ja, etwas	ja, wesentlich	ja, vollständig (Ausübung unmöglich)
☐	☐	☐	☐

5d.	Version 2: Beeinträchtigt Ihre Bewegungsstörung folgende Aktivitäten?		
Ankleiden			
nein	ja, etwas	ja, wesentlich	ja, vollständig (Ausübung unmöglich)
☐	☐	☐	☐

CVI : 1,0	KI:
**Umformulierung (Stammfrage)**<br>Berücksichtigter Kommentar:<br>• *„Aus meiner Sicht verständlicher: Beeinträchtigt ihre Bewegungsstörung …? Nein, etwas, wesentlich,…“*	**Unterstreichung (Stammfrage)**<br>• Unterstreichung unterstützt Betroffene, die Beeinträchtigung konsequent auf die Bewegungsstörung zu beziehen.

5d.	Version 1: Wie wirkt sich Ihre Bewegungsstörung auf folgende Alltagsaktivitäten aus?		
Körperpflege			
normal	etwas eingeschränkt	wesentlich eingeschränkt	unmöglich
☐	☐	☐	☐

5d.	Version 2: Beeinträchtigt Ihre Bewegungsstörung folgende Aktivitäten?		
Körperpflege			
nein	ja, etwas	ja, wesentlich	ja, vollständig (Ausübung unmöglich)
☐	☐	☐	☐

5d.	Version 2: Beeinträchtigt Ihre Bewegungsstörung folgende Aktivitäten?		
Körperpflege			
nein	ja, etwas	ja, wesentlich	ja, vollständig (Ausübung unmöglich)
☐	☐	☐	☐

CVI : 1,0	KI:
**Umformulierung (Stammfrage)**<br>Berücksichtigter Kommentar:<br>• *„Aus meiner Sicht verständlicher: Beeinträchtigt ihre Bewegungsstörung …? Nein, etwas, wesentlich,…“*	**Unterstreichung (Stammfrage)**<br>• Unterstreichung unterstützt Betroffene, die Beeinträchtigung konsequent auf die Bewegungsstörung zu beziehen.

6a.	Version 1:<br><br>**Wie wirken sich folgende Probleme auf Ihre Bewegungsfähigkeit aus?**		
	Schwindel oder Benommenheit		
	**kaum**	**mittel**	**stark**
	☐	☐	☐

6a.	Version 2:<br>**Fühlen Sie sich durch folgende Beschwerden beeinträchtigt, wenn Sie sich bewegen?**			
	Schwindel oder Benommenheit			
	**gar nicht**	**kaum**	**mittel**	**stark**
	☐	☐	☐	☐

6a.	Version 3:<br>**Fühlen Sie bei alltäglichen Bewegungen durch folgende Beschwerden beeinträchtigt?**				
	Schwindel oder Benommenheit				
	**gar nicht**	**kaum**	**mittel**	**stark**	**sehr stark**
	☐	☐	☐	☐	☐

CVI : 1,0
Umformulierung (Stammfrage)
Berücksichtigter Kommentar:
- *„Aus meiner Sicht klarere Info: Fühlen sie sich durch folgende Beschwerden beeinträchtigt: Gar nicht, kaum, mittel, stark“*

KI:
Umformulierung und Unterstreichung (Stammfrage)
- Ergänzung „alltäglich“ verdeutlicht den Kontext, in dem die Beeinträchtigung durch die Bewegungsstörung beurteilt werden soll.
- Unterstreichung unterstützt Betroffene, die Beeinträchtigung konsequent auf ihre Bewegungsfähigkeit zu beziehen.

Ergänzung (Antwortmöglichkeit)
- Ergänzung „sehr stark“ ermöglicht den Betroffenen eine weitere Differenzierung zwischen „stark“ und „sehr stark“.

6b.	Version 1:<br><br>**Wie wirken sich folgende Probleme auf Ihre Bewegungsfähigkeit aus?**		
	Schmerzen (z. B. Rückenschmerzen oder Gelenkschmerzen)		
	**kaum**	**mittel**	**stark**
	☐	☐	☐

6b.	Version 2:<br>**Fühlen Sie sich durch folgende Beschwerden beeinträchtigt, wenn Sie sich bewegen?**			
	Schmerzen (z. B. Rückenschmerzen oder Gelenkschmerzen)			
	**gar nicht**	**kaum**	**mittel**	**stark**
	☐	☐	☐	☐

6b.	Version 3:<br>**Fühlen Sie bei alltäglichen Bewegungen durch folgende Beschwerden beeinträchtigt?**				
	Schmerzen (z. B. Rückenschmerzen oder Gelenkschmerzen)				
	**gar nicht**	**kaum**	**mittel**	**stark**	**sehr stark**
	☐	☐	☐	☐	☐

**<u>CVI :</u> 1,0**<br>**Umformulierung (Stammfrage)**<br>Berücksichtigter Kommentar:<br>• *„Aus meiner Sicht klarere Info: Fühlen sie sich durch folgende Beschwerden beeinträchtigt: Gar nicht, kaum, mittel, stark“*	**<u>KI:</u>**<br>**Umformulierung und Unterstreichung (Stammfrage)**<br>• Ergänzung „alltäglich“ verdeutlicht den Kontext, in dem die Beeinträchtigung durch die Bewegungsstörung beurteilt werden soll.<br>• Unterstreichung unterstützt Betroffene, die Beeinträchtigung konsequent auf ihre Bewegungsfähigkeit zu beziehen.<br>**Ergänzung (Antwortmöglichkeit)**<br>• Ergänzung „sehr stark“ ermöglicht den Betroffenen eine weitere Differenzierung zwischen „stark“ und „sehr stark“.

6c.	**Version 1:**<br><br>**Wie wirken sich folgende Probleme auf Ihre Bewegungsfähigkeit aus?**	
	Empfindungsstörungen (z. B. Taubheit, Kribbeln, Missempfindungen)	
**kaum**	**mittel**	**stark**
☐	☐	☐

6c.	**Version 2:**<br>**Fühlen Sie sich durch folgende Beschwerden beeinträchtigt, wenn Sie sich bewegen?**		
	Empfindungsstörungen (z. B. Taubheit, Kribbeln, Missempfindungen)		
**gar nicht**	**kaum**	**mittel**	**stark**
☐	☐	☐	☐

6c.	**Version 3:**<br>**Fühlen Sie <u>bei alltäglichen Bewegungen</u> durch folgende Beschwerden beeinträchtigt?**			
	Empfindungsstörungen (z. B. Taubheit, Kribbeln, Missempfindungen)			
**gar nicht**	**kaum**	**mittel**	**stark**	**sehr stark**
☐	☐	☐	☐	☐

**<u>CVI :</u> 0,86**<br>**Umformulierung (Stammfrage)**<br>Berücksichtigter Kommentar:<br>• *„Aus meiner Sicht klarere Info: Fühlen sie sich durch folgende Beschwerden beeinträchtigt: Gar nicht, kaum, mittel, stark“*	**<u>KI:</u>**<br>**Umformulierung und Unterstreichung (Stammfrage)**<br>• Ergänzung „im Alltag“ verdeutlicht den Kontext, in dem die Beeinträchtigung durch die Bewegungsstörung beurteilt werden soll.<br>• Unterstreichung unterstützt Betroffene, die Beeinträchtigung konsequent auf ihre Bewegungsfähigkeit zu beziehen.<br>**Ergänzung (Antwortmöglichkeit)**<br>• Ergänzung „sehr stark“ ermöglicht den Betroffenen eine weitere Differenzierung zwischen „stark“ und „sehr stark“.

6f.	Version 1:<br><br>**Wie wirken sich folgende Probleme auf Ihre Bewegungsfähigkeit aus?**	6d.	Version 2:<br>**Fühlen Sie sich durch folgende Beschwerden beeinträchtigt, wenn Sie sich bewegen?**	6d.	Version 3:<br>**Fühlen Sie bei alltäglichen Bewegungen durch folgende Beschwerden beeinträchtigt?**
	Allgemeine körperliche Erschöpfung		Allgemeine körperliche Erschöpfung		Allgemeine körperliche Erschöpfung

kaum	mittel	stark
☐	☐	☐

gar nicht	kaum	mittel	stark
☐	☐	☐	☐

gar nicht	kaum	mittel	stark	sehr stark
☐	☐	☐	☐	☐

**CVI : 1,0**<br>**Umformulierung (Stammfrage)**<br>Berücksichtigter Kommentar:<br>• *„Aus meiner Sicht klarere Info: Fühlen sie sich durch folgende Beschwerden beeinträchtigt: Gar nicht, kaum, mittel, stark“*	**KI:**<br>**Umformulierung und Unterstreichung (Stammfrage)**<br>• Ergänzung „alltäglich“ verdeutlicht den Kontext, in dem die Beeinträchtigung durch die Bewegungsstörung beurteilt werden soll.<br>• Unterstreichung unterstützt Betroffene, die Beeinträchtigung konsequent auf ihre Bewegungsfähigkeit zu beziehen.<br>**Ergänzung (Antwortmöglichkeit)**<br>• Ergänzung „sehr stark“ ermöglicht den Betroffenen eine weitere Differenzierung zwischen „stark“ und „sehr stark“.

6g.	Version 1:<br><br>**Wie wirken sich folgende Probleme auf Ihre Bewegungsfähigkeit aus?**	6e.	Version 2:<br>**Fühlen Sie sich durch folgende Beschwerden beeinträchtigt, wenn Sie sich bewegen?**	6e.	Version 3:<br>**Fühlen Sie bei alltäglichen Bewegungen durch folgende Beschwerden beeinträchtigt?**
	Konzentrationsstörung oder geistige Umnebelung		Konzentrationsstörung		Konzentrationsstörung

kaum	mittel	stark
☐	☐	☐

gar nicht	kaum	mittel	stark
☐	☐	☐	☐

gar nicht	kaum	mittel	stark	sehr stark
☐	☐	☐	☐	☐

**CVI : 0,86**	**KI:**
**Umformulierung (Einzelitem und Stammfrage)**<br>Berücksichtigte Kommentare:<br>• *„Aus meiner Sicht klarere Info: Fühlen sie sich durch folgende Beschwerden beeinträchtigt: Gar nicht, kaum, mittel, stark“*<br>• *„Zwischen Benommenheit (6a) und geistiger Umnebelung zu differenzieren ist herausfordernd.“*	**Umformulierung und Unterstreichung (Stammfrage)**<br>• Ergänzung „alltäglich“ verdeutlicht den Kontext, in dem die Beeinträchtigung durch die Bewegungsstörung beurteilt werden soll.<br>• Unterstreichung unterstützt Betroffene, die Beeinträchtigung konsequent auf ihre Bewegungsfähigkeit zu beziehen.<br>**Ergänzung (Antwortmöglichkeit)**<br>• Ergänzung „sehr stark“ ermöglicht den Betroffenen eine weitere Differenzierung zwischen „stark“ und „sehr stark“.

Version 1:

**6h.**	**Wie wirken sich folgende Probleme auf Ihre Bewegungsfähigkeit aus?**		
	Angst zu stürzen oder mich zu verletzen		
	**kaum**	**mittel**	**stark**
	☐	☐	☐

Version 2:

**6f.**	**Fühlen Sie sich durch folgende Beschwerden beeinträchtigt, wenn Sie sich bewegen?**			
	Angst zu stürzen oder sich zu verletzen			
	**gar nicht**	**kaum**	**mittel**	**stark**
	☐	☐	☐	☐

Version 3:

**6f.**	**Fühlen Sie <u>bei alltäglichen Bewegungen</u> durch folgende Beschwerden beeinträchtigt?**				
	Angst zu stürzen oder sich zu verletzen				
	**gar nicht**	**kaum**	**mittel**	**stark**	**sehr stark**
	☐	☐	☐	☐	☐

**CVI : 1,0**	**KI:**
**Umformulierung (Stammfrage und Einzelitem)**<br>Berücksichtigte Kommentare:<br>• *„Aus meiner Sicht klarere Info: Fühlen sie sich durch folgende Beschwerden beeinträchtigt: Gar nicht, kaum, mittel, stark“*<br>• *„Angst zu stürzen oder sich zu verletzen?“*	**Umformulierung und Unterstreichung (Stammfrage)**<br>• Ergänzung „alltäglich“ verdeutlicht den Kontext, in dem die Beeinträchtigung durch die Bewegungsstörung beurteilt werden soll.<br>• Unterstreichung unterstützt Betroffene, die Beeinträchtigung konsequent auf ihre Bewegungsfähigkeit zu beziehen.<br>**Ergänzung (Antwortmöglichkeit)**<br>• Ergänzung „sehr stark“ ermöglicht den Betroffenen eine weitere Differenzierung zwischen „stark“ und „sehr stark“.

7a.	Version 1: Wie stark wirkt sich Ihre Bewegungsstörung auf folgende Bereiche des Lebens aus?		
Soziale Aktivitäten (Freunde, Familie)			
**Kaum**	**mittel**	**stark**	**Kann ich nicht sagen**
☐	☐	☐	☐

7a.	Version 2: Wie stark beeinträchtigt Ihre Bewegungsstörung folgende Bereiche Ihres Lebens?			
Soziale Aktivitäten (Freunde, Familie)				
**gar nicht**	**kaum**	**mittel**	**stark**	**trifft nicht zu**
☐	☐	☐	☐	☐

7a.	Version 3: Wie stark beeinträchtigt <u>Ihre Bewegungsstörung</u> folgende Bereiche Ihres Lebens?			
Soziale Aktivitäten (Freunde, Familie)				
**gar nicht**	**kaum**	**mittel**	**stark**	**trifft nicht zu**
☐	☐	☐	☐	☐

<u>CVI :</u> 1,0
Umformulierung (Stammfrage)
Berücksichtigte Kommentare:

- *„Beeinträchtigt?“*
- *„Kategorien: gar nicht, kaum, mittel, stark?“*

<u>KI:</u>
Unterstreichung (Stammfrage)

- Unterstreichung unterstützt Betroffene, die Beeinträchtigung konsequent auf die Bewegungsstörung zu beziehen.

7b.	Version 1: Wie stark wirkt sich Ihre Bewegungsstörung auf folgende Bereiche des Lebens aus?		
Partnerschaft und Intimität			
**Kaum**	**mittel**	**stark**	**Kann ich nicht sagen**
☐	☐	☐	☐

7b.	Version 2: Wie stark beeinträchtigt Ihre Bewegungsstörung folgende Bereiche Ihres Lebens?			
Partnerschaft und Intimität				
**gar nicht**	**kaum**	**mittel**	**stark**	**trifft nicht zu**
☐	☐	☐	☐	☐

7b.	Version 3: Wie stark beeinträchtigt <u>Ihre Bewegungsstörung</u> folgende Bereiche Ihres Lebens?			
Partnerschaft und Intimität				
**gar nicht**	**kaum**	**mittel**	**stark**	**trifft nicht zu**
☐	☐	☐	☐	☐

<u>CVI :</u> 1,0
Umformulierung (Stammfrage)
Berücksichtigte Kommentare:

- *„Beeinträchtigt?“*
- *„Kategorien: gar nicht, kaum, mittel, stark?“*

<u>KI:</u>
Unterstreichung (Stammfrage)

- Unterstreichung unterstützt Betroffene, die Beeinträchtigung konsequent auf die Bewegungsstörung zu beziehen.

7c.	Version 1:		
	Wie stark wirkt sich Ihre Bewegungsstörung auf folgende Bereiche des Lebens aus?		
	Freizeitgestaltung (Hobbies, Unterhaltung)		
**Kaum**	**mittel**	**stark**	**Kann ich nicht sagen**
☐	☐	☐	☐

7c.	Version 2: Wie stark beeinträchtigt Ihre Bewegungsstörung folgende Bereiche Ihres Lebens?			
	Freizeitgestaltung (Hobbies, Unterhaltung)			
**gar nicht**	**kaum**	**mittel**	**stark**	**trifft nicht zu**
☐	☐	☐	☐	☐

7c.	Version 3: Wie stark beeinträchtigt Ihre Bewegungsstörung folgende Bereiche Ihres Lebens?			
	Freizeitgestaltung (Hobbies, Unterhaltung)			
**gar nicht**	**kaum**	**mittel**	**stark**	**trifft nicht zu**
☐	☐	☐	☐	☐

CVI : 1,0
Umformulierung (Stammfrage)
Berücksichtigte Kommentare:
- *„Beeinträchtigt?“*
- *„Kategorien: gar nicht, kaum, mittel, stark?“*

KI:
Unterstreichung (Stammfrage)
- Unterstreichung unterstützt Betroffene, die Beeinträchtigung konsequent auf die Bewegungsstörung zu beziehen.

7d.	Version 1:		
	Wie stark wirkt sich Ihre Bewegungsstörung auf folgende Bereiche des Lebens aus?		
	Arbeit oder Ausbildung		
**Kaum**	**mittel**	**stark**	**Kann ich nicht sagen**
☐	☐	☐	☐

7d.	Version 2: Wie stark beeinträchtigt Ihre Bewegungsstörung folgende Bereiche Ihres Lebens?			
	Arbeit oder Ausbildung			
**gar nicht**	**kaum**	**mittel**	**stark**	**trifft nicht zu**
☐	☐	☐	☐	☐

7d.	Version 3: Wie stark beeinträchtigt Ihre Bewegungsstörung folgende Bereiche Ihres Lebens?			
	Arbeit oder Ausbildung			
**gar nicht**	**kaum**	**mittel**	**stark**	**trifft nicht zu**
☐	☐	☐	☐	☐

CVI : 1,0
Umformulierung (Stammfrage)
Berücksichtigte Kommentare:
- *„Beeinträchtigt?“*

KI:
Unterstreichung (Stammfrage)
- Unterstreichung unterstützt Betroffene, die Beeinträchtigung konsequent auf die Bewegungsstörung zu beziehen.

• *„Kategorien: gar nicht, kaum, mittel, stark?“*	

7f.	Version 1: Wie stark wirkt sich Ihre Bewegungsstörung auf folgende Bereiche des Lebens aus?		
Fürsorge (Kinder versorgen, Eltern pflegen)			
**Kaum**	**mittel**	**stark**	**Kann ich nicht sagen**
☐	☐	☐	☐

7e.	Version 2: Wie stark beeinträchtigt Ihre Bewegungsstörung folgende Bereiche Ihres Lebens?			
Fürsorge (Kinder versorgen, Eltern pflegen)				
**gar nicht**	**kaum**	**mittel**	**stark**	**trifft nicht zu**
☐	☐	☐	☐	☐

7e.	Version 3: Wie stark beeinträchtigt <u>Ihre Bewegungsstörung</u> folgende Bereiche Ihres Lebens?			
Fürsorge (Kinder versorgen, Eltern pflegen)				
**gar nicht**	**kaum**	**mittel**	**stark**	**trifft nicht zu**
☐	☐	☐	☐	☐

**<u>CVI :</u> 1,0**<br>**Umformulierung (Stammfrage)**<br>Berücksichtigte Kommentare:<br>• *„Beeinträchtigt?“*<br>• *„Kategorien: gar nicht, kaum, mittel, stark?“*	**<u>KI:</u>**<br>**Unterstreichung (Stammfrage)**<br>• Unterstreichung unterstützt Betroffene, die Beeinträchtigung konsequent auf die Bewegungsstörung zu beziehen.

7e.	Version 1: Wie stark wirkt sich Ihre Bewegungsstörung auf folgende Bereiche des Lebens aus?		
Transport (Autofahren, Bus und Bahn)			
**kaum**	**mittel**	**stark**	**Kann ich nicht sagen**
☐	☐	☐	☐

7f.	Version 2: Wie stark beeinträchtigt Ihre Bewegungsstörung folgende Bereiche Ihres Lebens?			
Autofahren				
**gar nicht**	**kaum**	**mittel**	**stark**	**trifft nicht zu**
☐	☐	☐	☐	☐

7f.	Version 3: Wie stark beeinträchtigt <u>Ihre Bewegungsstörung</u> folgende Bereiche Ihres Lebens?			
Autofahren				
**gar nicht**	**kaum**	**mittel**	**stark**	**trifft nicht zu**
☐	☐	☐	☐	☐

**CVI : 1,0**<br>**Umformulierung (Einzelitem und Stammfrage)**<br>Berücksichtigte Kommentare:<br>• *„Beeinträchtigt?"*<br>• *„Kategorien: gar nicht, kaum, mittel, stark?"*<br>**Itemsplitting**<br>Berücksichtigte Kommentare:<br>• *„aus meiner Sicht besser: Auto-, Bus- und Bahnfahren."*	**KI:**<br>**Unterstreichung (Stammfrage)**<br>• Unterstreichung unterstützt Betroffene, die Beeinträchtigung konsequent auf die Bewegungsstörung zu beziehen.

**7b.**	**Version 1:**<br><br>**Wie stark wirkt sich Ihre Bewegungsstörung auf folgende Bereiche des Lebens aus?**		
	Transport (Autofahren, Bus und Bahn)		
**kaum**	**mittel**	**stark**	**Kann ich nicht sagen**
☐	☐	☐	☐

**7g.**	**Version 2:**<br>**Wie stark beeinträchtigt Ihre Bewegungsstörung folgende Bereiche Ihres Lebens?**			
	Bus- oder Bahnfahren			
**gar nicht**	**kaum**	**mittel**	**stark**	**trifft nicht zu**
☐	☐	☐	☐	☐

**7g.**	**Version 3:**<br>**Wie stark beeinträchtigt <u>Ihre Bewegungsstörung</u> folgende Bereiche Ihres Lebens?**			
	Bus- oder Bahnfahren			
**gar nicht**	**kaum**	**mittel**	**stark**	**trifft nicht zu**
☐	☐	☐	☐	☐

**CVI : 1,0**<br>**Art der Veränderung:**<br>**Itemrevision Einzelitem und Stammfrage**<br>Berücksichtigte Kommentare:<br>• *„Beeinträchtigt?"*<br>• *„Kategorien: gar nicht, kaum, mittel, stark?"*<br>**Itemsplitting**<br>Berücksichtigte Kommentare:<br>• *„aus meiner Sicht besser: Auto-, Bus- und Bahnfahren."*	**KI:**<br>**Unterstreichung (Stammfrage)**<br>• Unterstreichung unterstützt Betroffene, die Beeinträchtigung konsequent auf die Bewegungsstörung zu beziehen.
