# Supplementary material for: Development and content validation of a questionnaire for functional movement disorders
Source: Nervenarzt. 2021 Dec 23;93(10):1009–18. [Article in German] doi: 10.1007/s00115-021-01247-1 (PMC9534974; doi:10.1007/s00115-021-01247-1)
Supplement: Supplementary file 4 [file 115_2021_1247_MOESM4_ESM.pdf]

Fragebogen für funktionelle Bewegungsstörungen

Dieser Fragebogen soll das Ausmaß und die Auswirkungen Ihrer Bewegungsstörung erfassen. Bitte beantworten Sie alle Fragen, indem Sie in jeder Reihe jeweils ein Kästchen markieren. Die Fragen beziehen sich auf Ihre Erfahrungen **in den letzten zwei Wochen**. Falls Ihre Beschwerden sehr wechselhaft sind, legen Sie Ihren Antworten einen typischen schlechten Tag innerhalb der letzten zwei Wochen zugrunde.

**1. Wie oft treten folgende Beschwerden auf?**	**nie**	**selten**	**manchmal**	**häufig**	**immer**
Muskelschwäche oder -lähmung	☐	☐	☐	☐	☐
Muskelverkrampfung	☐	☐	☐	☐	☐
Zittern oder Muskelzuckungen	☐	☐	☐	☐	☐
Unwillkürliche Bewegungen	☐	☐	☐	☐	☐
Ungezielte Bewegungen (Koordinationsstörungen)	☐	☐	☐	☐	☐
Bewegungsblockade oder -starre	☐	☐	☐	☐	☐

**2. Fühlen Sie sich im Alltag durch folgende Beschwerden beeinträchtigt, wenn Sie sich bewegen?**	**gar nicht**	**kaum**	**mittel**	**stark**	**sehr stark**
Muskelschwäche oder -lähmung	☐	☐	☐	☐	☐
Muskelverkrampfung	☐	☐	☐	☐	☐
Zittern oder Muskelzuckungen	☐	☐	☐	☐	☐
Unwillkürliche Bewegungen	☐	☐	☐	☐	☐
Ungezielte Bewegungen (Koordinationsstörungen)	☐	☐	☐	☐	☐
Bewegungsblockade oder -starre	☐	☐	☐	☐	☐

**3. Wie häufig sind folgende Körperteile von Ihrer Bewegungsstörung betroffen?**	**nie**	**selten**	**manchmal**	**häufig**	**immer**
Kopf/Hals	☐	☐	☐	☐	☐
Rumpf	☐	☐	☐	☐	☐
Arme und Hände	☐	☐	☐	☐	☐
Beine und Füße	☐	☐	☐	☐	☐

4.	Wie sicher können Sie folgende Bewegungen ausführen?	sicher	unsicher	nur mit Hilfsmittel möglich	nicht möglich, auch nicht mit Hilfsmittel
	Stehen	☐	☐	☐	☐
	Kurze Strecken gehen (< 10 m)	☐	☐	☐	☐
	Längere Strecken gehen (> 100 m)	☐	☐	☐	☐
	1 kg heben oder tragen	☐	☐	☐	☐

5.	Beeinträchtigt Ihre Bewegungsstörung folgende Aktivitäten?	nein	ja, etwas	ja, wesentlich	ja, vollständig (Ausübung unmöglich)
	Sprechen	☐	☐	☐	☐
	Essen und Trinken	☐	☐	☐	☐
	Schreiben oder Tippen	☐	☐	☐	☐
	Ankleiden	☐	☐	☐	☐
	Körperpflege	☐	☐	☐	☐

6.	Fühlen Sie sich bei alltäglichen Bewegungen durch folgende Beschwerden beeinträchtigt?	gar nicht	kaum	mittel	stark	sehr stark
	Schwindel oder Benommenheit	☐	☐	☐	☐	☐
	Schmerzen (z. B. Rückenschmerzen oder Gelenkschmerzen)	☐	☐	☐	☐	☐
	Empfindungsstörungen (z. B. Taubheit, Kribbeln, Missempfindungen)	☐	☐	☐	☐	☐
	Allgemeine körperliche Erschöpfung	☐	☐	☐	☐	☐
	Konzentrationsstörung	☐	☐	☐	☐	☐
	Angst zu stürzen oder sich zu verletzen	☐	☐	☐	☐	☐

7.	Wie stark beeinträchtigt Ihre Bewegungsstörung folgende Bereiche Ihres Lebens?	gar nicht	kaum	mittel	stark	sehr stark	trifft nicht zu
	Soziale Aktivitäten (Freunde, Familie)	☐	☐	☐	☐	☐	☐
	Partnerschaft und Intimität	☐	☐	☐	☐	☐	☐
	Freizeitgestaltung (Hobbies, Unterhaltung)	☐	☐	☐	☐	☐	☐
	Arbeit oder Ausbildung	☐	☐	☐	☐	☐	☐
	Fürsorge (Kinder versorgen, Eltern pflegen)	☐	☐	☐	☐	☐	☐
	Autofahren	☐	☐	☐	☐	☐	☐
	Bus- oder Bahnfahren	☐	☐	☐	☐	☐	☐
